# The relationship between the growth in the health sector and inbound health tourism: the case of Turkey

**DOI:** 10.1186/s40064-016-3341-8

**Published:** 2016-09-29

**Authors:** Harun Uçak

**Affiliations:** Department of Economics and Finance, Faculty of Business, Alanya Alaaddin Keykubat University, Alanya, Antalya, Turkey

**Keywords:** Health tourism, Health and social work expenditures, Granger causality, Johansen cointegration

## Abstract

One of the consequences of globalisation for Turkey, as well as in other emerging countries, has been an increasing trend in health tourism. Households have been considered choice the best option in terms of price and alternative possibilities while they have been solved their health problems. Previous studies have argued that the main drivers of the growth of inbound health tourism to developing countries are lower costs, shorter waiting periods, and better quality of care. This study aimed to test the effect of health and social service sector growth on the flow of inbound health tourism between 2004:Q1 and 2015:Q4 by employing Granger causality and Johansen cointegration approaches. Our findings suggested that there is a long-run Granger causality from domestic health and social work expenditures to health tourism income whereas this is non-existence in the opposite direction.

## Introduction

Health tourism has become one of the fastest growing subfields in the tourist sector in recent years. It has enhanced foreign exchange earnings, which has elevated economic opportunities in the destinations. Thus, an increasing number of countries have been competing for a greater market share and to encourage inbound tourism by presenting attractive opportunities in their medical services. These destinations have increased their investment in advanced technology and attractive accommodation (Horowitz et al. 2007). Furthermore, health care is predominantly a service industry and it has been affected by the liberalisation of the trade process that is related to the sector. Accordingly, the regulations of the World Trade Organization and its General Agreement on Trade in Services concerning the free movement of goods and services has accelerated the liberalisation of the trade in health services (Lunt et al. 2011).

In fact, health tourism is not a new type of economic field as people have always travelled in order to solve their health problems. In the past, wealthy people from less developed countries travelled to developed countries in order to access health services that were unavailable at home. Although travelling for medical care and well-being has long existed, Connell (2013) has indicated that there has been an increase in travelling from developed countries to developing or emerging countries for medical treatments. This occurs for a variety of reasons including cost considerations, shorter waiting periods, and better quality of care, among others. Hence, the direction of patient traffic has changed from being one directional to bi-directional. In the past, people in high income groups have travelled to developed countries to acquire advanced treatments, but middle-class people in developed countries have now begun to travel to developing countries to receive high-quality treatment with at a lower cost and this has become a new concept in the tourism sector (Chen and Flood 2013; Connell 2011; Horowitz and Rosensweig 2008; Ormond 2011).

Population dynamics should also be noted with respect to health tourism flow to developing countries. The proportion of elderly people within the total population, which is also known as an ageing population, has been a major problem in recent decades that has resulted in higher average spending on health care. Developed countries have been allocated greater financial resources for the health care of their citizens. According to OECD Health Statistics, health expenditure per capita is $8713 in the United States, $6325 in Switzerland, $5862 in Norway, $5130 in the Netherlands, $4818 in Germany (all of which area developed countries), and $1530 in Poland, $1048 in Mexico, and $941 in Turkey (developing countries). The data shows that the cost of health services is quite high in developed countries when compared with developing countries. However, aged populations share in developed countries is higher than other country groups in usual and public transfers are the main sources to cover costs (United Nations 2013). Considering that older people have higher average health expenditures than younger people, outbound health tourism or inbound skilled labour immigration may have positive macroeconomic consequences in terms of states’ budgets. Due to the rise in the world’s population and the rise in living standards and increases in health costs in developed countries, Lee and Hung (2010) has pointed out that the health care tourism sector in developing countries has been offering quality and comparatively affordable healthcare services through highly skilled personnel. This has increased foreign revenue in these developing countries, expanded job opportunities within the healthcare sector, and encouraged investors to invest more heavily in the health sector.

According to UNWTO (2016), 27 % of worldwide inbound travel includes visiting friends and relatives (VFR), religious reasons and pilgrimages, health treatment, and so on. Accordingly, OECD (2014a) indicated that there is significant potential for tourism growth in Turkey by combining historical, natural, and cultural heritage with health tourism.

The WHO Regional Office for Europe (2012) specified that Turkey is one of the few countries that has dramatically improved its health sector, achieving positive outcomes in a very limited time and, as such, has been considered as a successful example. According to the OECD (2014b) reports, there has been a significant amount of investment in the health sector with an increasing number of modern hospitals and the Health Transformation Programme (HTP) has also been established to complement these changes.

Access to health care services in another country is an important factor for health tourism market growth. Runnels and Carrera (2012) mentioned about accreditation of a relevant national agency to would be a significant contributor for quality of medical care. Turkey is accepted as a health tourism destination country, count among the states with one of the most number of JCI-accredited hospitals that are 49 in number currently.

Development plans that include sectoral plans have been done in Turkey as in many other countries. The current development plan is entitled The Tenth Development Plan 2014–2018 and it is noted that health expenditures are expected to increase and this process will encourage inbound medical tourism (T.R. Ministry of Development 2014). Furthermore, the importance of diversification of the product offer in the tourism sector has also been highlighted with a particular focus given to health tourism under the *health tourism improvement programme*, with the aim of increasing Turkey’s ranking in the global health tourism sector. With this aim in mind, it has been specifically noted that the institutional and legal infrastructure needs to be improved and cooperation between the public and the private sector should be strengthened.

The main objective of this study is to investigate the relationship between health sector growth and inbound tourism flow in Turkey. International organisations have viewed recent developments in the country’s health sector as successful and it is important to investigate whether it is this success that has led to the increase in inbound tourism.

## Background

The literature on health tourism has recently expanded, and it can be divided into three groups. The first group aims at defining health tourism and explaining its types. The second group of studies, which is mostly quantitative, tends to examine health tourism studies and typologies of health tourists, and to examine the motivations of health tourists or the reasons for preferring health tourism. The third group is empirical studies at the macro level that uses time series empirical attempts, but they have been limited due to series data availability.

Goodrich and Goodrich (1987) explored the concept of health tourism in one of the earliest academic studies in the field and indicated that it could be used to define an effective marketing strategy. More recently, increasing number of studies on health tourism and medical tourism has appeared in the tourism studies literature but most of these are limited with descriptive articles that explain the importance of health tourism. Pocock and Phua (2011) identified medical tourism using a conceptual framework to compare its policy implications for health systems in Malaysia, Thailand, and Singapore. Connell (2006) emphasised that medical tourism would continue to increase due to privatisation and cost differentials between countries. This author also pointed to the increasing trend of health tourism in Asia and the swift uptake in global interest has also been emphasised by Henderson (2004), Laing and Weiler (2008), Leng (2010), and NaRanong and NaRanong (2011).

There has also been also an increasing number of micro level survey studies in this field that aim to generalise findings on health tourism for use with higher levels of aggregation. Schalber and Peters (2012) examined a case study of health tourism in Alpine states. Using both quantitative and qualitative data, they investigated health tourism competitiveness in Alpine tourism from the perspectives of health tourism experts and the CEO’s of destination management organisations. For the case study, the authors used secondary sources the status of health tourism in the Alps is presented and serves as a starting point for the primary research. In order to operationalise the questionnaire they then used Porter’s model of national competitiveness (Porter, 1990), which was adapted by Smeral (1998), as a framework for the analysis. Sultana et al. (2014) measured the comparative importance of factors such as destination competitiveness, service quality, tourist attitude, and service cost from the perspectives of customers in India. The authors employed a survey equation modelling approach and a self-administered questionnaire that was administered to 235 international patients in order to collect the primary data. Based on data from 2002 to 2008, and from a case study that included variable comparison, Qi (2011) examined the relationship between medical tourism and the GATS (General Agreement on Trade in Services) commitments for Malaysia, Singapore, and Thailand. In the study’s methodology, visits of foreign patients were used as a dependent variable and GATS commitments were used as explanatory variables. Furthermore, the control variables that were applied were: countries’ health policy, delivery in private sector, human resources, cost of treatments, level of medical treatment services, language services, and democracy. Jónás-Berki et al. (2015) investigated the characteristics of health tourism destinations from a regional perspective in Hungarian and considered the direction of product development in health tourism, the seasonality and diversification of the product, and they asked a number of questions that included regional impact and quality orientation. Reddy et al. (2010) investigated the medical tourism beliefs of undergraduate students using the theory of planned behaviour. The findings show that educational intervention is important in helping to promote travel for medical treatment.

Despite the increasing amount of studies on health tourism, empirical research at the macro level has been limited. This may be because health tourism is a newly emerging tourism type and its contribution to the general economy cannot yet be analysed due to missing time series data and sectoral data. The empirical studies on tourism have largely generally focused on a country or region’s tourism sector as a whole. However, in recent decades developments of time series analysis methods and application of these methods, in parallel to the various fields of economics, have made efforts to investigate the contribution of tourism to economic growth, and also to test the tourism-led growth hypothesis. The literature shows that there have been many attempts to investigate the relationship between tourism and economic growth by using the methods of cointegration and causality tests. However, with limited time series empirical attempts for health tourism, Lee and Hung (2010) examined the causality between tourism, economic growth, and health care development. The authors used proxy variables of Gross Domestic Product (GDP) per capita for economic development, per capita government expenditure on health as a proxy for the development of the health care industry, and total tourist arrivals for tourism. Their findings for the short run show that there is a unidirectional causality from economic growth to health care development and a bidirectional causality from health care to tourism.

## Data and methodology

The article is based on quarterly time series data obtained from Turkish Statistical Institute (TURKSTAT) and the Central Bank of the Republic of Turkey (CBRT) that covers the period from 2004:Q1 to 2015:Q4. The GDP of health and social services (HGDP) is used as a proxy variable for economic growth of the health sector, which is reported in TRY constant prices. The second variable, inbound tourism flow (HTOUR), shows the tourism demand of international visitors to Turkey for the purpose of health or for medical reasons for a period of less than a year. The third variable is real exchange rate (RER) and it is used a proxy variable in order to test the effects of relative price changes. All variables are expressed in logarithm forms and seasonally adjusted. The following model was used to investigate the relationship between inbound tourism demand and health sector growth.

1 $$\ln htour_{t} = \alpha_{0} + \alpha_{1} \ln hgdp_{t} + \alpha_{2} \ln rer_{t} + \alpha_{3} u_{t}$$

In this study, the Granger causality test is employed to investigate causal relationships between health GDP, inbound health tourist flow, and real exchange rate. Granger introduced the concept of Granger causality in 1969 and it has been widely used in econometric studies to test availability and the direction of the causality (Granger 1969). Furthermore, Johansen’s cointegration analysis is employed to determine any long-term relationship between the variables before the causality test.

The first step in time series analysis is to investigate the stationarity of variables, also called the unit root test. Accordingly, the existence of a unit root at frequency zero would imply that the stochastic trend is non-stationary (Torraleja et al. 2009). Gujarati and Porter (2009) point out that it is so often to meet non-stationary time series and the estimates of non-stationary variables will lead to spurious regression. Thus, their economic interpretation will not be meaningful. Furthermore, unrelated time series may appear to be related using conventional testing applications such as ordinary least squares regression. To this end, we utilise the Augmented Dickey–Fuller test (ADF), and the Phillips–Perron test (PP) to examine whether the data are non-stationary (Dickey and Fuller 1981; Phillips and Perron 1988).

The augmented Dickey–Fuller (ADF) unit root test is one of the most accepted and widely used tests to investigate the stationarity of series (Park et al. 2016). The following equations are estimated for each of the time series (Gujarati and Porter 2009):

2 $$\Delta Y_{t} = \beta_{1} + \beta_{2} t + \delta_{0} Y_{t - 1} + \alpha_{i} \mathop \sum \limits_{i = 1}^{m} \Delta Y_{t - i} + \varepsilon_{t}$$

where $\Delta$ is the first difference operator, *t* is the time trend, *k* denotes the number of lags used, and *ɛ* is the error term. *β*, *δ* and *α* are parameters. The null hypothesis that series *Y*_*t*_ is non-stationary can be rejected if *δ*_0_ is statistically significant with negative sign (Huarng et al. 2006). In addition, *m* shows the optimal lag order, which is chosen carefully using the Schwarz criterion (AIC) in empirical method.

Another stationarity test, the Phillips–Perron (PP) unit root test, is a complementary feature of the ADF unit root test. The ADF test on the distribution of the error term is assumed to be statistically independent and constant variance. In the PP test, autocorrelation is considering changing the condition of the conditional variance of the error term that also means a more flexible assumption (Phillips and Perron 1988).

Regression analysis based on time series data implicitly assumes that the underlying data is stationary (Gujarati and Porter 2009) and it is usually the case that time series variables of macro economy are non-stationary. Alternatively, cointegration analysis allows for spurious results to be avoided by using non-stationary data, but all those series have to be integrated into the same order. Despite the range of different cointegration tests in the literature, the Engle–Granger (Engle and Granger 1987) and Johansen (1988, 1991) tests are widely used. In this study, the Johansen cointegration test is employed to test the existence of a long-run equilibrium relationship among the variables.

Following the suggestion of Granger (1988), the Granger causality test is implemented in the equations below:

3 $$\Delta \ln htour_{t} = \alpha_{1} + \sum\limits_{i = 1}^{p} {\beta_{1i} } \Delta \ln htour_{{t{ - }i}} + \sum\limits_{j = 1}^{q} {\beta_{2i} } \Delta \ln hgdp_{t - j} + \sum\limits_{l = 1}^{r} {\beta_{3i} } \Delta \ln rer_{t - i} + \varepsilon_{1t}$$

4 $$\Delta \ln hgdp_{t} = \alpha_{2} + \sum\limits_{i = 1}^{p} {\gamma_{1i} } \Delta \ln hgdp_{{t{ - }i}} + \sum\limits_{j = 1}^{q} {\gamma_{2i} } \Delta \ln htour_{t - i} + \sum\limits_{l = 1}^{r} {\gamma_{3i} } \Delta \ln rer_{t - i} + \varepsilon_{2t}$$

5 $$\Delta \ln rer_{t} = \alpha_{3} + \sum\limits_{i = 1}^{p} {\delta_{1i} } \Delta \ln rer_{{t{ - }i}} + \sum\limits_{j = 1}^{q} {\delta_{2i} } \Delta \ln htour_{t - i} + \sum\limits_{l = 1}^{r} {\delta_{3i} } \Delta \ln hgdp_{t - i} + \varepsilon_{3t}$$

where ln is the natural logarithm, $\Delta$ is the first difference operator, *p, q*, *r* denote the number of lagged variables, *ɛ*_*it*_ are error terms that are assumed to be normally distributed and white noise.

## Results

The main goals of the study are to investigate the relationship between sectoral growth and the inbound tourism sector for health, and to identify any possible direction of causality between them. Before conducting any causality test it is important to examine the stationary properties of the time series data. These were investigated using ADF and PP unit root tests for the three variables. The null hypothesis of the unit root test is as follows: H0 = unit root (non-stationary), and we can reject it when the p value is less than a specified significance level (0.01 or 0.05).

According to the results shown in Table 1, the ADF and PP unit root tests suggest that the null hypothesis of a unit root cannot be rejected for all variables at their level forms I(0), which show that they are non-stationary. However, the variables may be stationary after they are converted into the first difference. *∆*ln *htour, ∆*ln *hgdp* and *∆*ln *rer* show the first difference of the variables and it is found that all of them are stationary on this level, which is also called integrated of order one denoted as I(1).

**Table 1:** Unit root tests

Variables	Augmented Dickey–Fuller (ADF)		Phillips–Perron (PP)	
	Constant without trend	Constant with trend	Constant without trend	Constant with trend
Level				
ln *htour*	−2.049449	−2.764120	−1.974959	−2.718319
ln *hgdp*	−1.107769	−2.711389	−1.125967	−2.697990
ln *rer*	−2.024238	−2.470090	−2.159688	−2.571869
First difference				
∆ ln *htour*	−8.048863^a^	−7.948662^a^	−10.29624^a^	−13.54837^a^
∆ ln *hgdp*	−7.367436^a^	−7.518019^a^	−7.326674^a^	−7.469352^a^
∆ ln *rer*	−6.565184^a^	−6.649881^a^	−6.564661^a^	−6.654715^a^

^a^Indicate statistical significance at the 1 %, level

After obtaining evidence that the series used are integrated in the same order, I(1), the co-integration test was undertaken, based on Johansen and Juselius’s (1990) maximum likelihood framework. The null hypothesis to be tested is that there are no cointegration relationships, because an autoregressive model is sensitive to the selection of an appropriate lag length and it has to be conducted before cointegration. Table 2 shows the lag selection results under various criteria, such as Akaike, Schwarz, Hannan–Quinn’s sequential modified likelihood ratio (LR) and final prediction error. Lag order selection criteria suggest the consideration of one lag.

**Table 2:** VAR lag order selection criteria

Lag	LR	FPE	AIC	SC	HQ
0	NA	0.021414	4.669916	4.791565	4.715029
1	185.8321^a^	0.000310^a^	0.433203^a^	0.919800^a^	0.613657^a^
2	8.009741	0.000378	0.625815	1.477360	0.941609
3	15.97709	0.000362	0.564991	1.781484	1.016125
4	8.366230	0.000429	0.704204	2.285644	1.290678

*LR* sequential modified likelihood ratio (LR) test statistic (each test at 5 % level), *FPE* final prediction error, *AIC* Akaike information criterion, *SC* Schwarz information criterion, *HQ* Hannan–Quinn information criterion

^a^Indicates lag order selected by the criterion

After the stationary tests of the variables and lag selection, the cointegration analysis was employed to investigate the existence of any relationship between *∆*ln *htour, ∆*ln *hgdp* and *∆*ln *rer*. Table 3 shows the results of the cointegration test. As can be seen, the trace statistic and the maximum Eigen value statistic show the null hypothesis that there exists at most one cointegration vector cannot be rejected at 5 % level significance level. Consequently, there is a long-term equilibrium relationship between the variables that are considered in this study.

**Table 3:** Results from Johansen co-integration rank test (trace) and (maximum Eigen-value)

Hypothesized no. of CE(s)	Maximum Eigenvalue	Trace statistic	Max-Eigen statistic
None^a^	0.394513	32.08032 (29.79707)	23.07922 (21.13162)
At most 1	0.161570	9.001103 (15.49471)	8.106321 (14.26460)
At most 2	0.019264	0.894782 (3.841466)	0.894782 (3.841466)

Maximum Eigenvalue indicates 1 cointegration equation at the 5 %. Trace statistic indicates 1 cointegration equation at the 5 %

^a^Denotes rejection of the hypothesis at the 5 % level

Granger (1988) pointed out that if cointegration exists in a pair of I(1) series, there must be causation in at least one direction. Therefore, VAR Granger causality/block exogeneity Wald tests were applied to investigate the direction of causality among variables and the results are shown on Table 4. According to the findings, the χ2 statistics test rejects the null hypothesis of no Granger causality from ln *y* to ln *tour*, and indicates that there is unidirectional causality from health sector growth to inbound health tourist flow, at a 5 % level.

**Table 4:** VAR Granger causality/block exogeneity Wald tests

Excluded	Chi-sq χ2	df	Prob.
Dependent variable: ln *tour*			
ln *y*	4.514293	1	0.0336
ln *rer*	2.686789	1	0.1012
All	6.918476	2	0.0315
Dependent variable: ln *y*			
ln *tour*	0.037477	1	0.8465
ln *rer*	1.799414	1	0.1798
All	1.888176	2	0.3890
Dependent variable: ln *rer*			
ln *tour*	0.042433	1	0.8368
ln *y*	1.044803	1	0.3067
All	1.454149	2	0.4833

χ2 shows the Chi square statistic and df shows degrees of freedom

## Conclusion

The main result of the paper shows that sectoral growth of health services has encouraged inbound health tourism. Furthermore, it sends an important message to policy-makers about the developments of the health sector in providing potential as a service sector export. Another finding of the study is that there is no causality from number of health sector tourists to health sector expenditure in Turkey. This can be explained by the economic contribution of inbound health tourism to the whole sector, which is still limited. However, the increasing trend of inbound health tourism may provide a more important contribution in the years to come.

According to the UNWTO (2016), Turkey ranks 6th in arrivals and 12th in receipts in the international tourism market. Therefore, the number of tourists and the amount of income is inconsistent and, from an economic perspective, per capita inbound tourism income needs to be improved. Furthermore, diversification of tourism types and markets is crucial for the sustainability of the sector, which is sensitive to news media that might impact on mass tourism. However, the flexibility of health service provision is lower than that of entertainment activities. This means that if quality and price perceptions can be established for health services, they would be less affected than other type of tourism during periods of negative media.

As of 2015, the per capita expenditure of inbound tourists who pursued health issues is US$1773, whereas it was US$756 per capita for the Turkish tourism sector as a whole. Therefore, this figure is a strong motivation for policy-makers to encourage inbound tourist flow for health purposes in future years. The infrastructure development of the health sector can be expected to be a key determinant for inbound health tourism in terms of quality of services.

Finally, as shown by the literature, macro level empirical studies of health tourism have been limited. Probably, this is mostly a result of missing, or the lack of detailed time series about the sector. Thus, it might be expected that the number of country level causality analyses will be increased in the coming years.
